# Supplementary figures and images for: Probing the limits of cis-acting gene regulation using a model of allelic imbalance quantitative trait loci
Source: PLoS Genet. 2025 Apr 30;21(4):e1011446. doi: 10.1371/journal.pgen.1011446 (PMC12068699; doi:10.1371/journal.pgen.1011446)

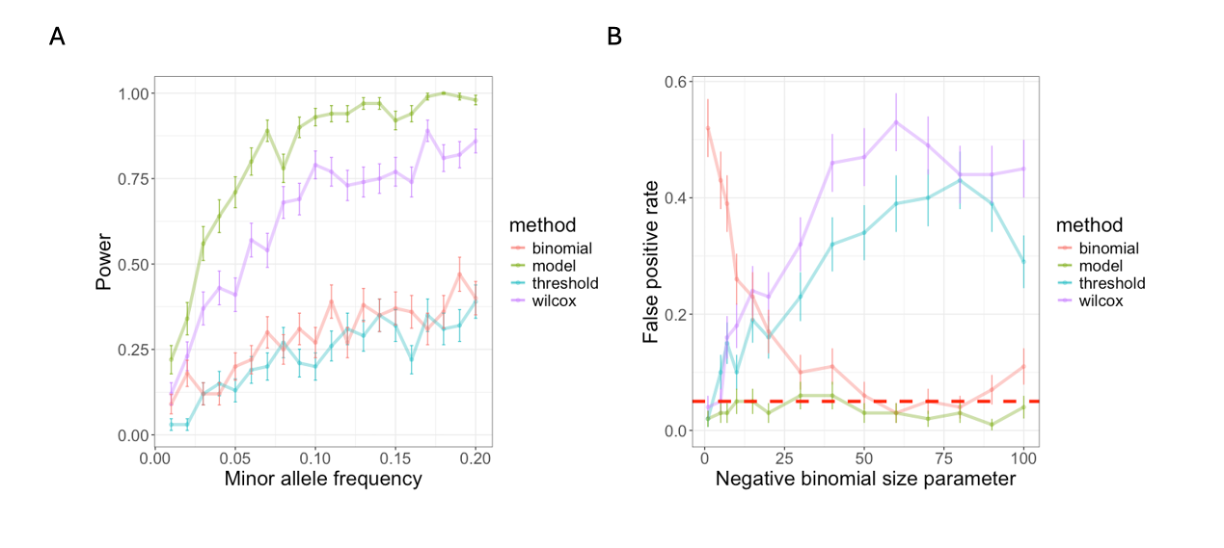

Supplement: S1 Fig — A) Comparison of the power of our method (referred to in the legend as ‘model’) and three other methods that can test for an association between ASE and the genotype of an eQTL (thereby inferring that the eQTL acts in cis). The results shown are based on the simulations illustrated in Fig 2A,B and correspond to an aiQTL of moderate effect size. B) False positive inferences of an aiQTL as a function of the size parameter of the negative binomial distribution. These results are based on simulations that are equivalent to those shown in Fig 1D, but with a trans-acting eQTL (resulting in an increase in gene expression by a factor of 1.3). The red dashed line shows the significance level of the tests (i.e. the expected false positive rate). (PNG) [file pgen.1011446.s001.png]

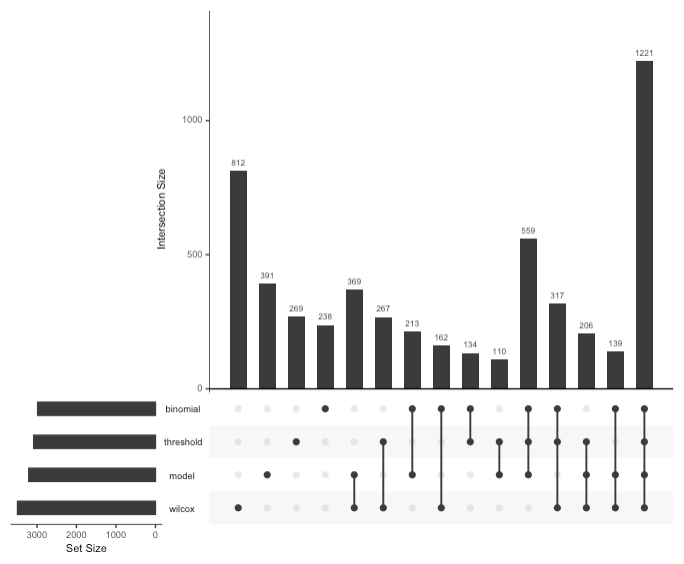

Supplement: S2 Fig — The strongest eQTL, with minor allele frequency of at least 0.05, was tested for each expressed gene in Whole Blood. Only cases where the eQTL could be tested using all four methods were considered. (PNG) [file pgen.1011446.s002.png]

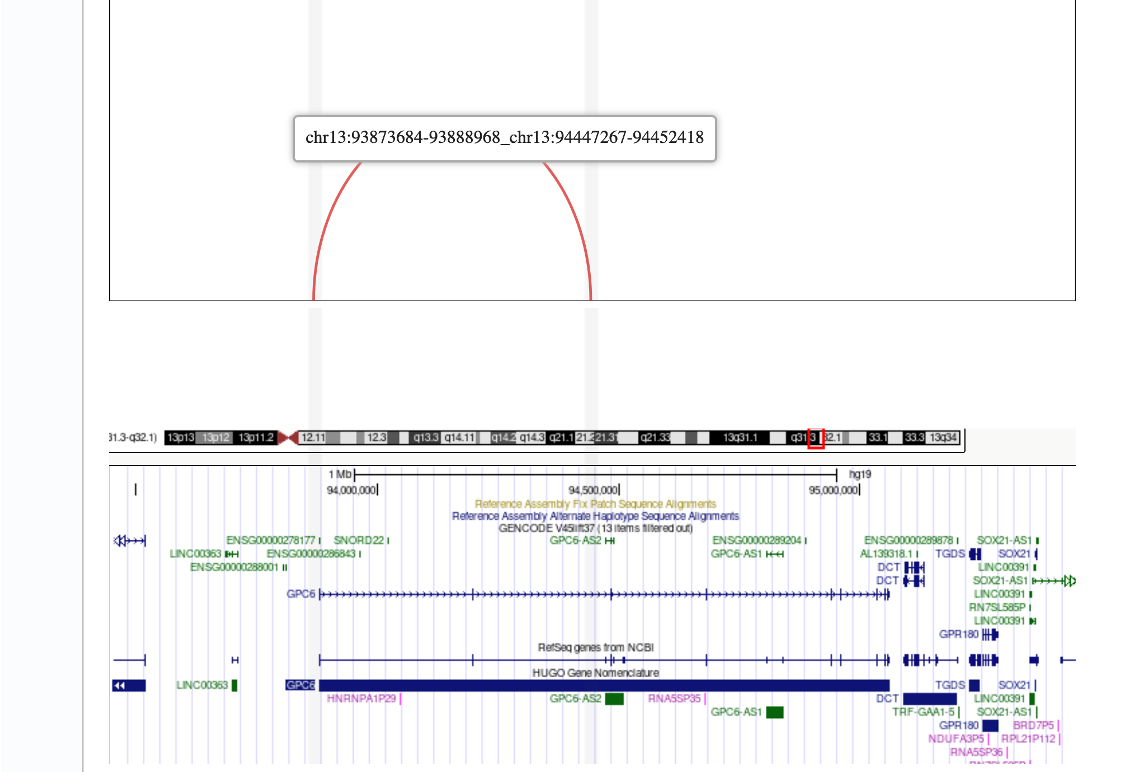

Supplement: S3 Fig — An example of a long-range aiQTL supported by a promoter Capture Hi-C data in the same tissue (aorta), generated using the 3D genome server (3dgenome.fsm.northwestern.edu/chic.php). The affected gene is GPC6, a glypican gene on chromosome 13 that is well over a megabase in length. The red arc indicates a chromatin interaction derived from the Capture Hi-C data that links the promoter region to the aiQTL, located in intron two of the gene and separated from the promoter by more than 570 kb. Coordinates shown are for the hg19 human genome assembly. (PNG) [file pgen.1011446.s003.png]

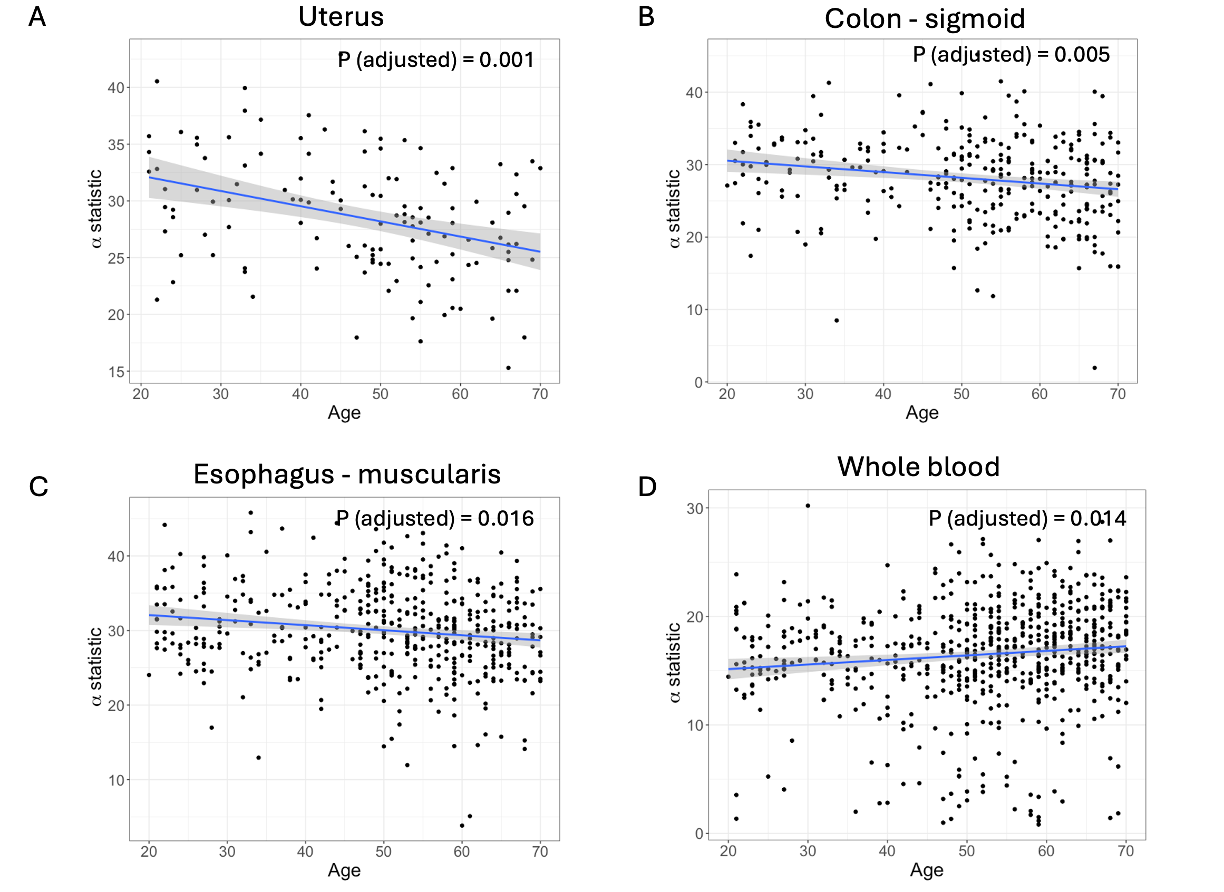

Supplement: S4 Fig — In the case of (A) Uterus, (B) Colon - sigmoid and (C) Esophagus - muscularis the α statistic showed weak negative correlated with age, consistent with a tendency towards increased allelic imbalance in older subjects. In Whole blood (D), the correlation was in the opposite direction. P values show within each of the panels have been adjusted for multiple testing using the Holm method. (PNG) [file pgen.1011446.s004.png]

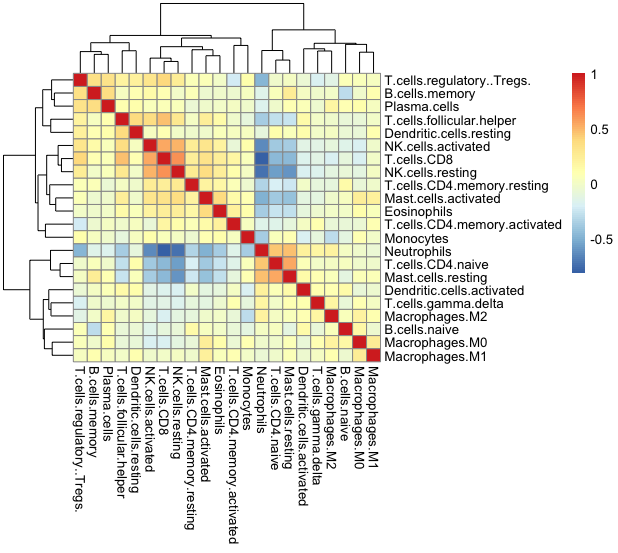

Supplement: S5 Fig — (PNG) [file pgen.1011446.s005.png]

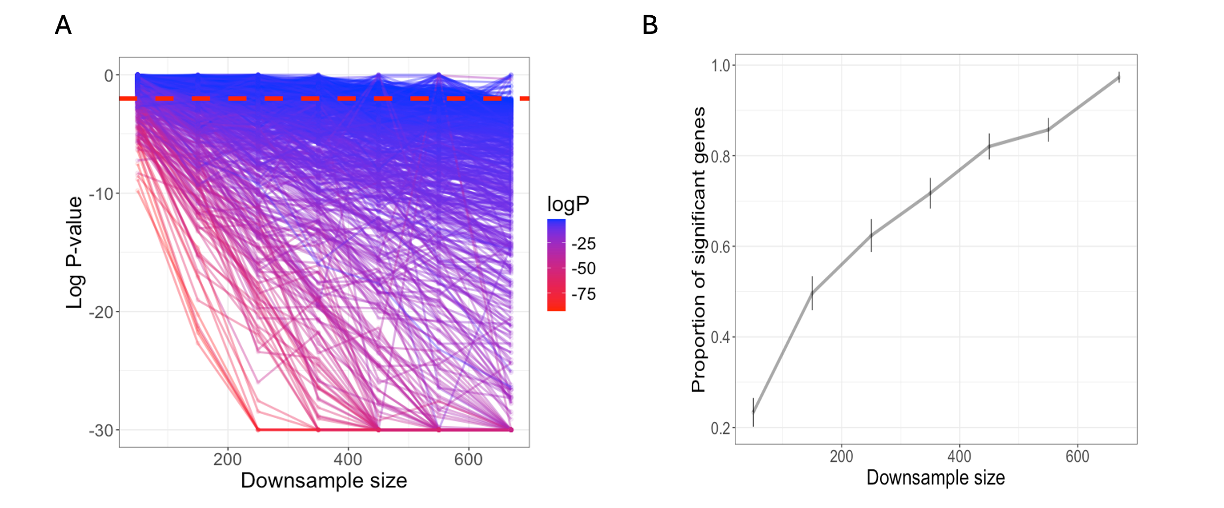

Supplement: S6 Fig — A) Log10 P-value of the aiQTL as a function of the downsampling sample size. Lines are coloured by the log10 P-value obtained using all 670 samples. The dashed line corresponds to the P-value of 0.01. The y-axis is truncated to a minimum log10 P-value of -30. B) The proportion of the aiQTLs that were detected, as a function of the downsampling sample size. Error bars show twice the standard error of the proportion (corresponding approximately to a 95% confidence interval). (PNG) [file pgen.1011446.s006.png]
