# Supplementary material for: Probing the limits of cis-acting gene regulation using a model of allelic imbalance quantitative trait loci
Source: PLoS Genet. 2025 Apr 30;21(4):e1011446. doi: 10.1371/journal.pgen.1011446 (PMC12068699; doi:10.1371/journal.pgen.1011446)
Supplement: Attachment — Submitted filename: Responses2Reviewers.docx [file pgen.1011446.s007.docx]

*Responses to reviewer comments are in italics*

**Reviewers' Comments**

Reviewer #1: In this manuscript, Seoighe et al. introduce a new computational method for identifying cis-acting genetic variation and apply it to GTEx data. The work is partially motivated by the important distinction between variants that are cis-acting versus simply located close to the gene. The method performs well in simulations, and its application to real data revealed that local eQTLs at a distance of more than 500 kb from the TSS of the affected gene are unlikely to act in cis – an interesting observation. Application to different tissues and estimated immune cell components are also good additions to the paper.
Overall, this is a strong, focused paper that does a nice job introducing a method that is a useful addition to the field. The paper is well written and the figures are clear. I do not have many comments.

*We are grateful to the reviewer for their positive assessment of our manuscript, highlighting the useful addition it provides to the field.*

My only somewhat major comment is that I wasn’t able to fully follow the description of the mixture model. Specifically, what are the two components? They appear not to be the two specific A and B alleles, since those are assigned arbitrarily in different samples. They also do not appear to be the homozygous and heterozygous samples since those are later compared using two estimates of π. So what are they? What do alpha1 and alpha2 refer to? A clarifying sentence or two should take care of this.

*The reviewer is correct that the two components do not correspond to the A and B alleles. We have revised our description of the model and have added a new figure to the revised manuscript that we hope makes our description easier to follow. The two components of our model are used to model variation in ASE between individuals. In a mixture model an observation can be thought of as having a certain probability of being derived from any of the mixture components. In the case of our model the data for an individual sample can be thought of as having some probability, π, of being an observation from the symmetric beta distribution with parameter alpha1 and probability 1- π of being an observation from the other component. E.g. if alpha1 is a low value (corresponding to a high level of imbalance) then π is the probability that the individual will have highly imbalanced expression (the proportion of reads that map to one of the alleles will be far from 0.5). Our tests evaluate whether these mixture probabilities differ significantly between samples that are heterozygous at the putative aiQTL compared to those that are homozygous at the putative aiQTL.*

Further, on page 9: “examples of enhances that act over large distances” should be “enhancers”.

*We thank the reviewer for pointing out this error, which we have corrected.*

Reviewer #2: Seoighe et al. have developed a new statistical method for analyzing allelic imbalance in gene expression and particularly genetic variants that contribute to this (aiQTLs). The method seems to provide some improvements over analogous prior methods, although systematic benchmarking is not provided. In addition to simulations, they apply the method to GTEx data, and describe how allelic imbalance changes as a function of variant distance to TSS, as well as overall as a function of age and cell type composition.

*[We have numbered the specific points raised by the reviewer to enable us to cross-reference our responses between points].*

1. While the paper has merits – the method seems fine and the analysis indicates some interesting findings – I find the presentation to be quite shallow. For a methods paper I’d need to see better benchmarking, stronger simulations, and overall more work to understand when and ho the method works and what its limitations are. As is, it’s difficult to be estimate whether it actually represents a step over state of the art. The analysis of GTEx data is fine, but the presentation and novelty of the results is somewhat lacking. I’m not even holding the bar at finding some truly novel phenomena, but not all the conclusions are well supported by the data provided, and/or additional steps could have bene taken for deeper insights.

*To address this point we have added a new section to the manuscript (‘Comparison to other methods’) with two new analyses. In the first we compare the power of our method to three other methods, one based on a binomial test, developed by Lefebvre et al., another based on using a fixed threshold on the allelic proportion and the third a method using a Wilcox rank sum test on the deviation from balanced expression, by Battle et al. We show a comparison of the power of our method and these methods as Supplementary Figure 1A. Our method has improved performance over these methods. In the second added analysis we demonstrate that all of these alternative methods are indeed biased, as we had argued (but not proven) in our original manuscript. The binomial test method is biased because the power to detect allelic imbalance depends on the total number of allele-specifically mapped reads and the total number of allele-specifically mapped reads, in turn, depends on gene expression. Because of this a trans-acting genetic variant that increases gene expression can result in an apparent association between ASE and the eQTL. The Battle et al. method is biased for a similar reason. This test compares the absolute value of the deviation from balanced expression (50% reads mapping to the reference allele) between heterozygotes and homozygotes of the eQTL. It is based on the absolute value of (r/(r+a) – 0.5), where r and a are the number of reads mapping to the reference and alternative alleles, respectively). However, for a gene with little or no imbalance, the absolute value of the deviation will tend to be smaller when the number of mapped reads is larger. A trans-acting eQTL, which has an impact on the total number of mapped reads, can again induce an apparent relationship between allelic imbalance and the eQTL when assessed using this metric. The method based on a fixed threshold for the allelic proportion is biased because samples with a smaller number of allele-specifically mapped reads can deviate more from 0.5 by chance. The results that demonstrate these biases are in Supplementary Figure 1B of the revised manuscript.*

*We provide a response to the key point about whether the conclusions drawn from our analysis of the GTEx data are well supported in response to the reviewer’s specific points below.*

2. I also have some specific comments, in no particular order of importance:
- coloc-eQTLs / colloc-eQTL (both spellings occur) – I would suggest another name.

*Thank you for pointing out this inconsistency. We agree that our terminology was not optimal and have replaced coloc-eQTL with proximal eQTL in the revised manuscript. We feel that this term conveys the idea of an eQTL that is located close to the gene with which it is associated.*

3. Page 2 intro on using ASE to identify rare variants – some references might be in order here?

*References have been added in the revised manuscript to support this point.*

4. “linkage disequilibrium between the eQTL and affected gene” – you probably mean “between the eQTL and variants in the affected gene”

*Correct. This has been amended.*

5. “our results suggest that the majority of eQTLs between half a megabase and one megabase from the eGene do not act in cis.” This is a strong and extraordinary claim, contrary to biological expectations. It is not sufficiently supported by the data provided. Rather, I think that the results presented are fully compatible with the known weaker cis-regulatory effects of variants at larger distances from TSS (due to weaker but real enhancer effects), which leads to lower power to detect them as aiQTLs.

*This is a key point.*

*First, we contend that the result should not be seen as extraordinary. It is consistent with a result in the GTEx main paper from 2017, which the reviewer mentions in a later point. However, the result in the GTEx paper used a method that required linkage disequilibrium to be modelled and is potentially affected by uncertainty in phasing, which increases with distance. Our paper provides a way to investigate this without considering linkage disequilibrium.*

*Secondly, our result is not compatible with the known weaker cis-regulatory effects of variants at larger distances from TSS. This is a crucial point. The logistic regression models we used to investigate the relationship between the distance from the TSS and the probability that an eQTL acts in cis included the log-transformed p-value for the*

*eQTL association as a covariate. This was stated in the Methods and Results sections. This model provides evidence that, for a given strength of association, the probability that an eQTL is also an aiQTL (and therefore inferred to act in cis) declines rapidly with distance from the gene (as illustrated in Fig. 3b of the revised manuscript).*

*We feel that it is important for the field to have an insight into the relationship between distance from the TSS and the probability that an eQTL acts in cis. The rapid decline of the probability of acting in cis with distance is well supported by our analysis.*

6. The simulations assume full lack of LD between the aiQTL and the transcribed variant. Could the authors simulate situations with partial LD, since this is often going to be the case? This may lead to loss of power when not all genotype classes are observed at randomly expected frequencies

*The method itself makes no assumption of LD but is also applicable when there is LD. The only situation in which linkage can result in loss of power is when it causes failure to perform allele-specific mapping of sequencing reads for one of the putative aiQTL genotypic groups (homozygotes or heterozygotes). In such a case no method will have power to associate allelic imbalance with the putative aiQTL. We note, however, that any effect of LD on power should not contribute to the result concerning the relationship between distance and the probability of acting in cis. Differences in power to detect ASE were accounted for in our logistic regression models (by including both the number of individuals with allele-specifically mapped reads and the total number of allele-specifically mapped reads as covariates in the model).*

7. The fold change effect sizes are quite strong (1.2) even for the weakest simulated effects. It would be good to have more simulations and/or e.g. downsampling of empirical data to test how properties of the data affect the performance of the method.

*We would like to note that these are expression fold changes and not log fold changes nor standardized effect sizes. Therefore, 1.2 corresponds to an eQTL that increases the expression level by 20%, which is not an unrealistically strong effect size in our experience. Already it is clear that at this effect size that our method (and those to which we have now compared it) begin to struggle to identify aiQTLs, with typical values of the other simulation parameters. It is likely that with the data analyzed here we will not detect aiQTLs with weaker effects. This is consistent with the fact (as noted by the reviewer in point 9, below) that even at the TSS we only detect just over 50% of the eQTLs as aiQTLs. We do not suggest that for an individual eQTL the lack of a detected aiQTL effect means that it acts in* trans. *We have added to our description of this result (on page 10 of the revised manuscript) to provide a more detailed explanation of this.* *Also, to explore this further, we have added the downsampling analysis suggested by the reviewer (new Supplementary Figure 6 in the revised manuscript). This also illustrates that there are likely to be many aiQTLs that remain to be detected, because the proportion of aiQTLs continues to rise as the number of samples approaches the full size of the data. As noted in the Discussion section, this is in-part because only a proportion of the data can contribute to the aiQTL signals (allele-specifically mapped reads), meaning that for some variants an eQTL will be detectable but an aiQTL will not (i.e. it will not be possible to infer that the variant acts in cis). Increased read lengths associated with long-read sequencing technologies and larger sample sizes should lead to increased power to detect aiQTLs.*

8. It’ not true that the extent of allelic imbalance for cis-eQTLs as the function of distance from TSS hasn’t been tested before; see GTEx main paper 2017. The results are somewhat corroborative (although see next point), but this should be referenced and described

*We believe the result the reviewer is referring to is in one of the supplementary figures of the 2017 GTEx paper. We have been unable to find complete information from that paper on how this result was generated; however, we agree it is important to reference this paper and have included it where this result is introduced in the revised manuscript. We have also modified the text of our abstract to remove the claim that this is the first time that the relationship between allelic imbalance and distance from the TSS has been examined. We note also that the GTEx result required phased data (inferred from population phasing in the paper) whereas our method does not require phased data.*

9. I don’t think I quite understand the model or the data behind Figure 2b. Writing down the actual equation would be helpful. It seems to me that samples with just 2 allelic reads are included but at that level the data is extremely sparse. I’m not sure I understand this right, but if from this logistic model one derives coefficients to then plot the probability of Fig 2a for the given parameters, then the result shown there is extremely specific to this set of parameters and not really biologically interpretable across the parameter space. If the y-axis is to be taken with biological interpretation of “suggest that direct cis-acting effects at a distance of 1Mbp up or downstream of the TSS are rare”, I note that the probability is only 0.5 even at TSS – and it would be a highly implausible claim that half of eQTLs at TSS don’t act in cis.

*We used standard logistic regression models to investigate the relationship between multiple features and the probability that an eQTL was detected as an aiQTL. Although this was stated in the Data and Methods section and in the legend of the figure (Figure 3 of the revised manuscript), we did not state it in Results (we have done so in the revised manuscript). The model included additive fixed effects only and all of the predictors are provided in Data and Methods.*

*Our mixture model differs from earlier methods in that it can make efficient use of sparse data. We do not perform e.g. binomial tests to detect ASE within an individual. Instead, our mixture model detects a tendency over a collection of individuals for individuals who are heterozygous at the putative aiQTL to tend to display greater allelic imbalance than homozygous individuals. In this context, even an individual with two allele-specifically mapped reads can contribute some (albeit a small amount) of evidence (because these two reads can be derived from the same or from different alleles – they are more likely to be derived from the same allele if there is allelic imbalance).*

*We do not claim that only 50% of the eQTLs at the TSS act in cis. Figure 3b (revised manuscript) shows the probability of detecting a significant aiQTL associated with the eQTL as a function of distance from the TSS (and for the values of the other parameters indicated in the figure legend). I.e. the probability of rejecting the null hypothesis that the mixture weights are the same for the homozygotes and heterozygotes of the eQTL. We will, of course, not detect 100% of the aiQTLs. We have revised this section of the manuscript to explain this point more clearly and added to it to tease out the implications of the TSS-distance term in the logistic model. If we assume that 100% of the eQTLs at the TSS act in cis, then we show that the fit of our logistic regression model implies that at 500 kb from the TSS only around 28% of the eQTLs act in cis. At 1Mb the proportion of eQTLs we detected as aiQTLs was no longer greater than the significance level of our test.*

10. The point above raises the question: if/since allelic analysis is often underpowered to find evidence of even a true cis-effect, isn’t it the case that this analysis can support an eQTL being in cis, but absence of evidence doesn’t mean evidence of absence? If this is the case, can we actually use the approach described here for the purpose of determining whether an eQTL acts in cis or not?

*We agree with the reviewer that a single test, with no further analysis, would not provide evidence for the absence of a cis effect. However, absence of evidence can provide evidence of absence when test power is taken into account. We included the factors that influence the power of the test in our logistic regression models for this reason. The factors that affect our capacity to detect an aiQTL are the strength of evidence for the eQTL, the number of samples for which allele-specifically mapped reads are available, the median number of allele-specifically mapped reads per sample and the minor allele frequency of the eQTL. All of these were modeled in our logistic regression models. These models suggest that when power to detect aiQTLs is taken into account, the distance from the TSS remains strongly associate with whether evidence that an eQTL is an aiQTL will be detected. We were not able to find sufficient methodological information for the earlier result (GTEx, 2017) that presages this result to determine whether power to detect ASE was adequately modeled in that case.*

11. The LM22 leukocyte reference might not be that great for whole blood deconvolution, as the GTEx whole blood contains other cell types than leukocytes; particularly hemoglobin expression is very high.

*Several previous studies have used LM22 for gene expression deconvolution of whole blood (e.g. PMID: 38306997, 37391799, 37202447). It is important to note that in our case, the inferred cell type proportions lead to a null result. We find that the correlation between the extent of allelic imbalance in whole blood and age disappears when we included the inferred cell type proportions in the statistical models. Our interest was in the relationship between age and allelic imbalance. Even if the deconvolution has picked up on some other trend in the gene expression data, the fact that changes in gene expression can explain the relationship between age and allelic imbalance suggests that this is not a general tendency for allelic imbalance to increase with age, but rather a feature of the genes that were relatively more or less highly expressed in the older individuals. In the revised manuscript we have modified our claims with this in mind. For example, instead of stating that the correlation of allelic imbalance and age is likely due to changes in cell type proportions, we say that it may be due to variation in blood cell composition. The study of variation in allelic imbalance across cell types need not be restricted to blood cells. A comprehensive analysis of this would be a significant undertaking and would require sorted cells and/or single cell data from samples for which genotype data is also available (see response to point 14).*

12. Note that the GTEx LCLs have a high degree of clonality (see e.g. Baral et al. 2015 Genome Research) which will lead to allelic imbalance from random monoallelic expression. This explains the result for the LCLs in Fig 3a.

*We agree and have added this explanation to the section “Allelic imbalance across samples and tissues” in the revised manuscript. We could not find the paper by Baral et al. in 2015 in Genome Research, but an earlier paper (Plagnol et al. 2008; PMID: 18698422) also makes this point, specifically highlighting its relevance for studies of ASE. We have added a reference to that paper in the revised manuscript.*

13. Isn’t clonal hematopoiesis a potential reason for reduced allelic imbalance in blood, associated with age and cell type composition?

*Clonal hematopoiesis could potentially make a contribution; however, clonal hematopoiesis is a strong function of age. We did not find an association with age when we corrected for cell type proportions.*

14. I have a feeling that more could be done on the cell type specificity of random monoallelic expression that is cites as the likely main cause of the differences in allelic imbalance. The references to prior literature seem shallow. Isn’t there accessible data (RNA-seq from sorted blood cell populations) that would allow testing some of the hypotheses that are posed?

*We agree that RNA-Seq from sorted blood cell populations is available; however, our method requires genotype data as well as gene expression data. While it is possible to recover genotypes from RNA-Seq, this introduces a strong bias in the case of our analysis, because allele-specific expression directly impacts on the probability that a heterozygous variant is detected. Evaluating variation in the extent of imbalance between specific cell types could be an interesting topic for future work, given availability of the appropriate data. This need not be restricted to blood cell types and identification of specific cell populations or pathways within cell types that show greater allelic imbalance (i.e. lower alpha using our model) could be informative. Doing this across all cell types could be informative, but would be a substantial undertaking and is beyond the scope of this paper.*

15. The section “Allelic imbalance across samples and tissues” describes a lot of results that are not shown in any display item. It would be interesting to see these, as main figure panels or in the supplement.

*Thank you for the suggestion. We have added a supplementary figure to the revised manuscript, illustrating the relationship between allelic imbalance and age (Supplementary Figure 4 of the revised manuscript). As mentioned in the text, three of the tissues show a weak negative correlation between the alpha statistic and age (consistent with a tendency for increased allelic imbalance with age). The only tissue with a significant (following multiple test correction) correlation in the opposite direction was Whole Blood.*

16. In general, the figure panels should include plotted color legends rather than having these described in the legend text; the current style is very difficult to decipher. It’s also extremely confusing when the meaning of colors switches between panels, e.g. in Fig 1 red is sometimes weak, sometimes strong effect.

*There was an error in the colours of this figure (Figure 2 in the revised manuscript). We apologise for the confusion this would have caused and have amended it in the revised manuscript. We thank the reviewer for highlighting this issue. The colours are now consistent across panels (though their precise interpretation is slightly different between panel D, compared to B and C, which is why we feel it is necessary to explain this in the legend text, rather than putting colour legends on the figure panels).*

17. Fig 1a: I don’t understand the x-axis label (and consequently, the entirely figure).

*This is the log-likelihood ratio test statistic (twice the difference in log likelihood). It has been provided in full in the revised manuscript.*

18. Fig 1b: Do I understand correctly that strong ASE is linked to lower power? How, why?

*We thank the reviewer for highlighting this issue (in Figure 2 of the revised manuscript). There was an error in the colours here (the same error referenced in response to point 16), which we have corrected in the revised manuscript. In both panels strong ASE is linked to higher power, as would be expected. We thank the reviewer for drawing our attention to this and apologise for the confusion that resulted from the error.*

19. “Genetic drift in regulatory regions could result in an increased tendency towards expression imbalance in individuals with a higher degree of heterozygosity” I fail to see how genetic drift (in the exact definition of the phrase) has a role here. Functional genetic variation can arise from a number of population genetic processes, and nothing in the analyses here addresses these.

*By genetic drift here, we were referring to the accumulation of random genetic changes that are not driven by selection; however, we agree with the reviewer that there was no need to reference genetic drift specifically here and we have removed it from the revised manuscript.*

Reviewer #3: In this paper, the authors propose a statistical model for identifying aiQTLs to investigate the limits of cis-acting gene regulation. Their model, based on a symmetric beta-binomial distribution, is designed to detect cis-regulatory eQTLs without relying on linkage disequilibrium, making it particularly effective for identifying long-range regulatory effects. Their methods are validated using both simulated data and real data from the GTEx consortium. The advantages of these methods include their robustness against false positives, improved power to detect cis-acting eQTLs even at large genomic distances, and adaptability for tissues with varying gene expression levels. Overall, this approach can be a very valuable tool for mapping regulatory variants across the genome. The manuscript is well-organized, although the clarity of the presentation could be significantly improved.

*We thank the reviewer for their positive assessment of the value of our approach and the quality of the manuscript.*

1. The "Model and Implementation" section is too brief and lacks technical details. For example, descriptions of the data (not individual datasets, but the form and setting of the data) and overall settings of the problem are absent. There should be an explicit description of the number of samples, the A allele, etc. Ideally, a diagram or graph should be included to illustrate the form of the data. This diagram should display the data, the problem to be solved, and the differences in assumptions made by their methods compared to previous methods. Additionally, the authors need to provide the log-likelihood function of the model, which was used to estimate the parameters, and clearly mathmatically formulate the null and alternative hypotheses.

*We thank the reviewer for the suggestion and have extended the description of our model and added a new figure (Figure 1 in the revised manuscript) to help improve clarity. The extended model description includes the formula for the full log-likelihood of the model. This allowed us to explain more clearly the null and alternative hypotheses. For the revised manuscript we have also added a link to a GitHub repository that includes an R implementation of the model as well as some example data. All of these changes are in Data and Methods (Model and Implementation).*

2. In the same section, the explanation of why setting alpha = beta is unclear, although briefly mentioned. When alpha = beta, the beta distribution always has a mean of 1/2; what implications does this have (for the symmetry of the beta-binomial distribution?)? Is this assumption necessary to align with biological facts? If such an assumption arises purely to control the number of parameters used, could it compromise the biological interpretation or even complict with the biological fact?

*This is the crucial aspect of our model that enables it to model long-range cis-acting eQTLs without the requirement of linkage disequilibrium. We focus on modeling imbalance rather than on modeling the direction of imbalance. Suppose we randomly label one allele at a heterozygous site within the transcript (i.e. a site within an exon of the eGene) as the A allele. The true (but unknown) proportion of reads from the A allele is a random variable. The expected value of this random variable will indeed be 0.5 (because the A allele is designated randomly). The alpha parameter essentially controls how far this random variable can stray from 0.5. This is how we model ASE. We hope that the extended description of the model and additional figure have helped to make this clearer.*

3. It is commendable that the authors simulated data that do not exactly follow the assumed distribution, which greatly aids in evaluating the robustness of the proposed model. However, in the simulated data, the authors did not compare the proposed method with existing methods or other regression/mixture models, making it difficult to determine whether the achieved power is high.

*We thank the reviewer for this suggestion (also suggested by Reviewer 2) and have added this comparison to the revised manuscript in a new section (Comparison to other methods) and a new figure (Supplementary Figure 1) in the revised manuscript.*

4. Similarly, in real data analyses, a detailed comparison with existing methods and an explanation of why their methods are superior are needed. The comparison should include a Venn diagram-like illustration of the agreement/difference in findings from different methods, as well as a detailed analysis of those differences--which findings make more sense?

*In the original manuscript we suggested that the alternative methods have the potential for bias. This is because these methods first define a measure of ASE at the eGene (using a binomial test, a Wilcoxon test or a fixed threshold; see response to Reviewer 2 point 1) and then compare this measure between groups of individuals, defined by the genotype at the eQTL. We suggested that this could induce a bias because by altering the expression level of the eGene a trans-eQTL can alter the power of the initial test or metric to detect ASE (which may have other genetic or non-genetic causes). This could result in a false positive inference of a cis effect. In the revised manuscript we demonstrate using simulations that trans-eQTLs can, indeed, be incorrectly identified by these methods as acting in cis (new Supplementary Figure 1 in the revised manuscript). The same simulations show that our model (which does not require an initial binary determination of ASE) is not subject to this bias. Following the reviewer’s suggestion, we have also added a new supplementary Figure to the manuscript comparing our results on real data (Whole Blood from GTEx) to those obtained by other methods (Supplementary Figure 2 in the revised manuscript). We have used an upset plot in place of a Venn diagram as these are easier to interpret. On the real data, the largest number of aiQTLs was detected by the Wilcox method (followed by our model); however, the Wilcox method is subject to systematic bias, as shown in Supplementary Figure 1 and these additional putative aiQTLs may well be false positives.*
